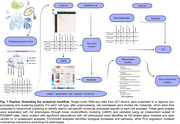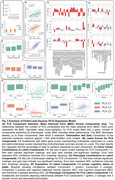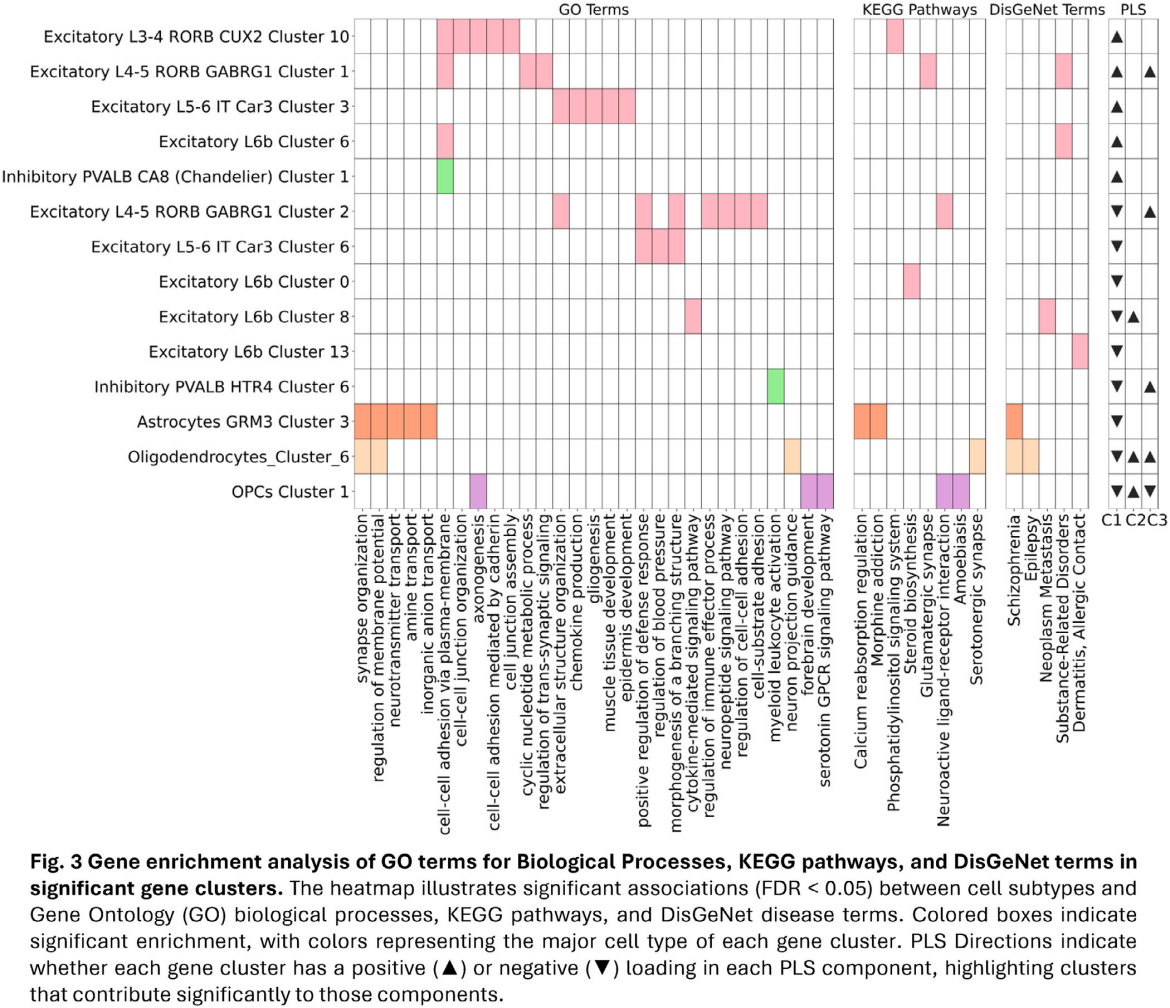# Coordinated multi‐cellular responses to Alzheimer's disease pathology reveal sex‐specific resilience signatures

**DOI:** 10.1002/alz70855_104816

**Published:** 2025-12-24

**Authors:** Gabriele Vilkaite, Lijun An, Yu Xiao, Ines Hristovska, Chris Gaiteri, Alexa Pichet Binette, Niklas Mattsson‐Carlgren, Jacob W. Vogel

**Affiliations:** ^1^ Department of Clinical Sciences Malmö, SciLifeLab, Lund Univerisity, Lund, Sweden; ^2^ Clinical Memory Research Unit, Department of Clinical Sciences Malmö, Faculty of Medicine, Lund University, Lund, Sweden; ^3^ Department of Psychiatry, Upstate Medical University, Syracuse, NY, USA; ^4^ Rush Alzheimer's Disease Center, Rush University Medical Center, Chicago, IL, USA; ^5^ Université de Montréal, Montréal, QC, Canada; ^6^ Centre de Recherche de l’Institut Universitaire de Gériatrie de Montréal, Montréal, QC, Canada; ^7^ Memory Clinic, Skåne University Hospital, Malmö, Skåne, Sweden; ^8^ Department of Clinical Sciences Malmö, SciLifeLab, Lund University, Lund, Sweden

## Abstract

**Background:**

Alzheimer's disease (AD) triggers multicellular transcriptomic responses that may reflect and moderate disease progression. While most studies observe these likely‐concurrent cell‐specific changes independently, the current study investigates multi‐cellular associations with AD phenotypes.

**Method:**

We analyzed single‐nuclei transcriptomics from the dorsolateral prefrontal cortex (DLPFC) of 427 donors (ROSMAP, Mathys et al.; 2.3M cells). Metacells were generated using k‐nearest neighbor clustering, and hierarchical gene clustering identified cell‐type‐specific gene modules (Figure 1). AD‐related gene modules (AGMs) were identified as clusters that showed cross‐validated associations with any disease phenotypes. AGMs underwent gene‐set enrichment analysis and were entered into a partial least squares (PLS) analysis, deriving multicellular interactions simultaneously predicting multiple AD neuropathological measures and clinical features.

**Result:**

We identified 28 AGMs across 11 cell subtypes. Cross‐validation of PLS regression led to selection of three components, while permutation testing confirmed the model's non‐random structure (Figure 2A). Component 1 reflected a combinatorial effect of all significant AD‐related gene clusters responding to increased pathology, being strongly associated with amyloid‐β (Aβ), tau and TDP‐43 pathology, and negatively with APOE E2 carriage (Figure 2B‐F). Components 2 (men) and 3 (women) highlighted potential sex‐specific resilience responses, positively associated with age and Aβ but negatively with tau pathology (Figure 2B‐F). Gene‐set enrichment (Figure 3) revealed Component 1 to involve downregulation of defense responses and upregulation of cell junction and adhesion across vulnerable neuronal types, perhaps indicating movement away from defense and toward glia‐mediated self‐destructive processes. Enrichment further suggested that components 2 and 3 involved oligodendrocyte‐mediated synaptic remodeling and neuronal immune/defense responses, with component 2 (men) involving L6B excitatory neurons and component 3 (women) more involving RORB‐GABRG excitatory and PVALB‐HTR4 inhibitory neurons. Astrocytes and oligodendrocyte precursor cells contributed opposing loadings relative to each other in components 2 and 3.

**Conclusion:**

Multiple cell types exhibit concurrent shifts, suggesting potential multi‐cellular associations with AD pathology. Changes in the first component reflected a generalized response to neurodegenerative pathology, likely representing neuronal death processes of selective neuronal subpopulations. In contrast, two additional multicellular responses emerged, suggesting sex‐specific cellular activity moderating resilience to AD pathology. Coordinated cell‐type‐specific alterations underscore the need to address cross‐cellular interactions in AD.